# Characterization of *HSP70* family in watermelon (*Citrullus lanatus*): identification, structure, evolution, and potential function in response to ABA, cold and drought stress

**DOI:** 10.3389/fgene.2023.1201535

**Published:** 2023-05-31

**Authors:** Xinsheng Wang, Zhi Jin, Yina Ding, Meng Guo

**Affiliations:** ^1^ School of Wine and Horticulture, Ningxia University, Yinchuan, China; ^2^ Key Laboratory of Modern Molecular Breeding for Dominant and Special Crops in Ningxia, Yinchuan, China; ^3^ Ningxia Modern Facility Horticulture Engineering Technology Research Center, Yinchuan, Ningxia, China; ^4^ Ningxia Facility Horticulture Technology Innovation Center, Ningxia University, Yinchuan, China

**Keywords:** heat shock proteins, genome-wide analysis, expression pattern, ABA, abiotic stress

## Abstract

Watermelon (*Citrullus lanatus*) as a crop with important economic value, is widely cultivated around the world. The heat shock protein 70 (HSP70) family in plant is indispensable under stress conditions. However, no comprehensive analysis of watermelon *HSP70* family is reported to date. In this study, 12 *ClHSP70* genes were identified from watermelon, which were unevenly located in 7 out of 11 chromosomes and divided into three subfamilies. ClHSP70 proteins were predicted to be localized primarily in cytoplasm, chloroplast, and endoplasmic reticulum. Two pairs of segmental repeats and 1 pair of tandem repeats existed in *ClHSP70* genes, and *ClHSP70s* underwent strong purification selection. There were many abscisic acid (ABA) and abiotic stress response elements in *ClHSP70* promoters. Additionally, the transcriptional levels of *ClHSP70s* in roots, stems, true leaves, and cotyledons were also analyzed. Some of *ClHSP70* genes were also strongly induced by ABA. Furthermore, *ClHSP70s* also had different degrees of response to drought and cold stress. The above data indicate that *ClHSP70s* may be participated in growth and development, signal transduction and abiotic stress response, laying a foundation for further analysis of the function of ClHSP70s in biological processes.

## 1 Introduction

With the acceleration of industrialization and global warming, plants are suffering from various abiotic stresses, including drought, salinity, heat, and low temperature (Zhang et al., 2022). Plants have developed a variety of regulatory mechanisms to cope with stresses during the long course of evolution, one of which involves heat shock proteins (HSPs). HSPs protect plant cells from stresses by folding and translocating new proteins and refolding denatured proteins (Dong et al., 2001; Timperio et al., 2008; Mohamed, 2011). Based on the molecular masses, HSPs can be divided into five subfamilies, including small HSP (sHSP), chaperonin (HSP60), 70-kDa-heat shock protein (HSP70), HSP90 and HSP100 subfamily (Wang et al., 2004). Among them, plant HSP70s are highly conserved and widespread, which are crucial housekeeping proteins, suggesting that HSP70s play essential roles under normal and stress conditions (Tanya and Elizabeth, 2007; Saeed et al., 2019).

Since the first discovery and identification in the early 1960s (Ritossa, 1996), great progress has been made in the study of HSP70s. HSP70 proteins consist of three conserved domains, an N-terminal ATPase domain (NBD, ∼44 kDa), a substrate binding domain (SBD, ∼18 kDa), and a C-terminal substrate binding domain (∼10 kDa) (Dragovic et al., 2006). According to different cell localization, plant HSP70s can be divided into four functional subfamilies: cytoplasm, endoplasmic reticulum (ER), plastid, and mitochondria (Guy and Li, 1998; Cho and Choi, 2009). Intracellular HSP70s were induced by heat stress to transfer to plasma membrane and liquid membrane, formed peripheral proteins outside the membrane, inhibited membrane protein degeneration, and ultimately improved biofilm stability and heat resistance (Jiang et al., 2021). Ectopic expression of cytoplasmic *CaHSP70-2* from pepper improved heat tolerance by altering the expression of stress-related genes in transgenic *Arabidopsis* (Guo et al., 2016). *Arabidopsis thaliana* also showed diurnal variation in heat tolerance, with the highest tolerance in the daytime, which was positively correlated with the expression levels of *AtHSP70s* (Dickinson et al., 2018). Besides, the expression of *HSP70s* was associated with drought, virus, heavy metal, and cold stress (Queitsch et al., 2000; Fei et al., 2015; Zhang et al., 2015; Osman et al., 2019; Lubkowska et al., 2021). For instance, overexpression of *Nicotiana tabacum NtHSP70-1* not only improved the plants’ thermoprotective activity but also enhanced drought tolerance (Cho and Hong, 2006). Cytoplasmic *HSP70s* could enhance the resistance of *Nicotiana benthamiana* to diverse viral infections (Chen et al., 2008). *Aquatic Moss HSP70s* might be related to detoxification of heavy metals, such as lead, cadmium, copper, and zinc (Esposito et al., 2012). However, as this family is multigenic, there may be functional redundancy among plant HSP70 members.

Watermelon (*Citrullus lanatus*) as an important horticultural crop with high economic and nutritional value, is widely cultivated in the world. It not only provides rich carbohydrates and dietary fiber, but also provides a variety of vitamins and compounds that are beneficial to human health (Makaepea et al., 2019; Mashilo et al., 2022). However, watermelon is sensitive to adverse conditions, and HSP70 proteins may play an important role in this response process. Members of the HSP70 family have been identified in different species, such as *Arabidopsis* (Lin et al., 2001), tobacco (Song et al., 2019), herbaceous peony (Zhao et al., 2019), sorghum (Nagaraju et al., 2020) and rice (Jung et al., 2013), but not in watermelon. The completion of genome sequencing of watermelon is helpful to identify its HSP70 family (Guo et al., 2013).

In this study, a total of 12 *ClHSP70* genes were identified in watermelon. The molecular characteristics, gene structure, *cis*-acting elements, SSRs, GO enrichment analysis, and protein interactions of ClHSP70 members were further analyzed, as well as chromosome mapping, phylogenetic tree construction, interspecific collinearity analysis and binding sites targeted by miRNAs. In addition, the tissue specificity of *ClHSP70s* was analyzed by qRT-PCR, and their response to ABA, drought and cold stress was also discussed. These results help us to better understand the function of *ClHSP70s* and provide key clues to reveal the response mechanism of watermelon to ABA and abiotic stresses.

## 2 Materials and methods

### 2.1 Plant materials and treatments

The watermelon variety ‘Xi Nong 8’ was used in this study. Seedlings were cultured in a light incubator at day/night 28/22°C (16/8 h) for 30 days, and Hoagland nutrient solution was used for hydroponics. For tissue expression analysis, the roots, stems, true leaves and cotyledons of seedlings were sampled. The seedlings placed in the Hoagland nutrient solution containing 10% polyethylene glycol 6000 (PEG6000) were used for drought treatment, the seedlings under 4°C conditions were used for cold treatment, and the seedlings spraying 100 μM ABA (dissolved in 0.004% ethanol) for hormone treatment. The control seedlings for ABA treatment were sprayed with 0.004% ethanol, and the control for abiotic stresses were the hydroponic seedlings under normal conditions. The leaves were collected after treatment for 0, 0.5 (cold and drought stress), 1, 3, 6, 12, and 24 h. Three biological replicates were performed for each treatment, and samples for each replicate were collected from five seedlings.

### 2.2 Identification of *ClHSP70* genes from watermelon

The identification process of ClHSP70 family in watermelon was shown in Supplementary Figure S1. Briefly, the watermelon genome sequence was downloaded from *EnsemblPlants* database (EB, http://plants.ensembl.org/info/data/index.html). The Hidden Markov Model (HMM) profile of HSP70 domain (PF00012) was downloaded from Protein family (Pfam) database (Finn et al., 2014) and used as a BLAST query against the watermelon genome database (E-value<1 × 10^−5^). Meanwhile, HMMER-3.0 program was employed to identify watermelon ClHSP70s (Finn et al., 2011). Subsequently, SMART (http://smart.embl-heidelberg.de/) and Pfam (http://pfam.xfam.org/search) were used to identify the HSP70 domains in all putative ClHSP70s to verify whether they belonged to HSP70 family.

The chromosome distributions, amino acid (AA), genome sequences and gene structures of watermelon *ClHSP70* members were obtained from EB database. In addition, EXPASY Proteomics Server (https://web.expasy.org/protparam/) was used to analyze the AAs, molecular mass, and theoretical isoelectric points (Lopes-Caiter et al., 2013). The cell localization prediction of ClHSP70s was performed using Wolf PSORT program (http://wolfpsort.org/).

### 2.3 Phylogenetic analysis

The protein sequences of HSP70 family from various species, including *A. thaliana*, *Solanum lycopersicum*, *Solanum tuberosum*, *Csativus sativus*, *Vitis vinifera*, *Zea mays*, *Oryza sativa* and *C. lanatus*, were multiply aligned using the ClustalW program of MEGA11 (Tamura et al., 2021). The gene IDs of *HSP70* family from different plants were shown in Supplementary Table S1. The phylogenetic tree was constructed based on the maximum likelihood (ML) method with 1000 bootstrap replications using TBtools (Chen et al., 2020).

### 2.4 Prediction of conserved motifs and three-dimensional structures and gene structure analysis

Conserved motifs in ClHSP70 sequences were searched by MEME suite (https://meme-suite.org/meme/) (Bailey et al., 2009), with the following parameters: any number of repetitions; maximum number: 10; optimum widths: 6-200 AAs. SWISS-Model (https://swissmodel.expasy.org/) was used to model three-dimensional (3D) structures of ClHSP70s based on homology modeling (Waterhouse et al., 2018). Global model quality estimation (GMQE) was used to evaluate the model quality (Wang et al., 2023). To illustrate the exon/intron structures of all *ClHSP70s*, the cDNA sequences were aligned with their genomic sequences via TBtools.

### 2.5 Chromosomal localization, synteny analysis and gene duplication

The chromosomal localizations of *ClHSP70s* and the genome files of watermelon, rice and *Arabidopsis* were obtained from EB database. The chromosome distribution and gene duplication of *ClHSP70* genes were analyzed and mapped using TBtools software, which was also used to visualize the collinearity map between different species. The duplication of *ClHSP70s* was defined according to the following criteria: a) the sequence alignment length covered more than 70% of the longer gene sequence; b) more than 70% similarity between aligned gene regions (Gu et al., 2002; Yang et al., 2008). Tandem duplicated genes were defined as two genes separated by no more than five genes in a 100 kb chromosomal region (Wang et al., 2010). Paralogous genes located on duplicated chromosomal blocks were considered as segmental duplication (Wei et al., 2007).

The ratios of nonsynonymous (Ka) and synonymous (Ks) (Ka/Ks) were calculated for the results of *ClHSP70* duplications, and Ks was converted into the differentiation time (T) with the formula: T = Ks/(2 × 6.1×10^−9^) ×10^−6^ million years ago (Mya) (Lynch and Conery, 2000).

### 2.6 Analysis of *cis*-regulatory elements and protein interaction

The promoter sequences of *ClHSP70s* (upstream sequence 2,000 bp) were identified by TBtools. The *cis*-elements were analyzed by PlantCARE online (https://bioinformatics.psb.ugent.be/webtools/plantcare/html/). The interaction analysis of ClHSP70s was performed using the STRING online (http://cn.string-db.org/), and *Arabidopsis* was used as a model plant for the query. The results were exported and plotted using Cystoscope software.

### 2.7 GO enrichment analysis and SSR mining of *ClHSP70s*

Gene Ontology (GO) enrichment analysis of watermelon *ClHSP70s* was conducted using AmiGO 2 (http://amigo.geneontology.org/amigo/landing) with the corrected *p*-value < 0.05. The GO analysis contains cellular component (CC), biological process (BP), and molecular function (MF).

The sequences of 12 *ClHSP70s* were downloaded from the watermelon genome, and the bases were set as 2,000 bp before the start codon and 2,000 bp after the stop codon. These sequences were subjected to simple sequence repeat (SSR) mining by MISA (https://webblast.ipk-gatersleben.de/misa/index.php?action=1) (Beier et al., 2017). The minimum standard lengths for dinucleotide, trinucleotide and tetranucleotide repeats were 6, 5 and 5 repeats, respectively (Fu et al., 2023).

### 2.8 Response of *ClHSP70s* to cold and drought stress based on RNA-seq data

Fragments Per Kilobase Per Million (FPKM) values for each *ClHSP70s* in leaves under cold stress (4°C for 36 h, PRJNA328189) and drought stress conditions (4 days and 8 days, PRJNA454040) were retrieved from CuGenDB database (http://cucurbitgenomics.org/r naseq/home). Watermelon cultivar Y134 was used for cold treatment, while other two varieties Y34 and M20 were used for drought stress treatment. The data for each *ClHSP70* were normalized by log2 (FPKM) **(** Supplementary Table S2 **)** and shown as Heatmap using TBtools.

### 2.9 RNA extraction and RT-qPCR

Total RNA was extracted from samples by Total RNA kit (TIANGEN, Beijing, China), and reverse-transcribed by One-Step gDNA Removal and cDNA Synthesis Super Mix kit (TransGen, Beijing, China) according to the manufacturer’s instructions. The final cDNA was used as the template for subsequent quantitative real-time PCR (qRT-PCR).

Specific primers for detection of *ClHSP70* gene expression (Supplementary Table S3) were designed using Primer 5 software. The yellow-leaf-specific proein8 (*ClYLS8*) and *β*-actin (*ClACT*) genes from watermelon were used as the reference genes (Kong et al., 2014). qRT-PCR was conducted using ChamQ universal SYBR qPCR Master Mix (Vazyme, Nanjing, China) with a 20 μL system on the qTOWER³ Series-Real-Time Thermal Cyclers (Analytik Jena, Thüringen, Germany). The experimental procedure of qRT-PCR carried out in accordance with the instruction manual of SYBR qPCR Master Mix, including an initial activation of 95°C for 30 s, then 40 cycles of 95°C for 10 s, 60°C for 30 s. The 2^−△△CT^ method was used to calculate the relative transcript levels of *ClHSP70s* (Schmittgen and Livek, 2008).

### 2.10 Prediction of binding sites of *ClHSP70s* targeted by miRNA

The microRNA (miRNA) sequences were downloaded from PmiREN2.0 (https://www.pmiren.com/) (Guo et al., 2022). The prediction of binding sites of *ClHSP70s* targeted by miRNA was performed using psRNATarget (https://www.zhaolab.org/psRNATarget/) (Dai et al., 2018), and the parameters were set to default values. The track predicted *ClHSP70* gene binding sites were listed in Supplementary Table S4.

## 3 Results

### 3.1 Identification and characterization of watermelon *HSP70* family members

Using HMMER search and BLASTP alignment, 12 *HSP70* family members were finally identified (Supplementary Table S5). They were named *ClHSP70-1* (62.01 kDa) to *ClHSP70-12* (102.80 kDa) based on their molecular weight. The physicochemical properties of ClHSP70s were analyzed by EXPASY online software. The *ClHSP70* genes were unevenly distributed on 7 of 11 chromosomes. The minimum length of the encoding sequence was 1,716 bp and the maximum length was 2,766 bp, the number of AAs corresponding to the encoding proteins was 571 and 921, respectively (Table 1). The theoretical isoelectric points were between 4.9 (ClHSP70-6) and 5.78 (ClHSP70-7), indicating that ClHSP70 proteins were acidic. Only the total average hydrophilicity (GRAVY) of *ClHSP70-1* was greater than 0, indicating that most ClHSP70s were hydrophilic proteins. Six members (*ClHSP70-1, -2, -3, -4, -5,* and *-11)* were mainly located in cytoplasm, one (*ClHSP70-10*) in nucleus, two (*ClHSP70-6* and -9) in chloroplasts, one (*ClHSP70-7*) in mitochondria, and two (*ClHSP70-8* and -12) in endoplasmic reticulum.

**TABLE 1:** The detailed information of *ClHSP70* members.

Gene name	ID	Chr	Genomic position	CDS	Exon	AA	MW	PI	GRAVY	Subcellular localization
*ClHSP70-1*	Cla97C05G086420.1	5	4793613–4795328	1716	0	571	62.07	5.55	0.011	cyto: 8, chlo: 4, nucl: 1, cysk: 1
*ClHSP70-2*	Cla97C04G075000.1	4	22595298–22597953	1944	1	647	70.86	5.16	−0.403	cyto: 7, cysk: 5, chlo: 1, nucl: 1
*ClHSP70-3*	Cla97C09G181360.1	9	34779571–34781996	1947	1	648	70.98	5.16	−0.409	cyto: 8, cysk: 4, chlo: 1, nucl: 1
*ClHSP70-4*	Cla97C11G217390.1	11	21467157–21469382	1941	1	646	71.08	5.15	−0.411	cyto: 11, chlo: 2, nucl: 1
*ClHSP70-5*	Cla97C09G181410.1	9	34816934–34819290	1959	1	652	71.51	5.1	−0.439	cyto: 8, cysk: 4, chlo: 2
*ClHSP70-6*	Cla97C09G172860.1	9	9437336–9441818	2034	6	677	72.94	4.9	−0.293	chlo: 8, mito: 5, nucl: 1
*ClHSP70-7*	Cla97C10G192810.1	10	20737655–20741060	2043	5	680	73.04	5.78	−0.309	mito: 12, chlo: 2
*ClHSP70-8*	Cla97C01G001870.1	1	1690732–1693969	1998	7	665	73.46	5.11	−0.46	E.R. 14
*ClHSP70-9*	Cla97C06G119280.1	6	18182550–18186176	2118	7	705	75.39	5.27	−0.293	chlo: 14
*ClHSP70-10*	Cla97C05G095550.1	5	22168414–22173677	2289	8	762	85.39	5.69	−0.456	nucl: 12, cyto: 1, vacu: 1
*ClHSP70-11*	Cla97C09G164300.1	9	1853201–1857386	2535	8	844	92.73	5.37	−0.397	cyto: 8, nucl: 3, chlo: 1, cysk: 1, E.R. 1
*ClHSP70-12*	Cla97C11G222680.1	11	28722840–28732590	2766	14	921	102.80	5.29	−0.494	E.R. 14

Chr, Chromosome; CDS, length of coding sequence; AA, number of amino acids; MW, molecular weight, KDa; PI, theoretical isoelectric point; GRAVY, grand average of hydropathicity. Nucl, nucleus; Mito, mitochondria; Chlo, chloroplast; Cyto, cytosol; E.R., endoplasmic reticulum; Cysk, cytoskeleton; Vacu, vacuole.

**FIGURE 1:**
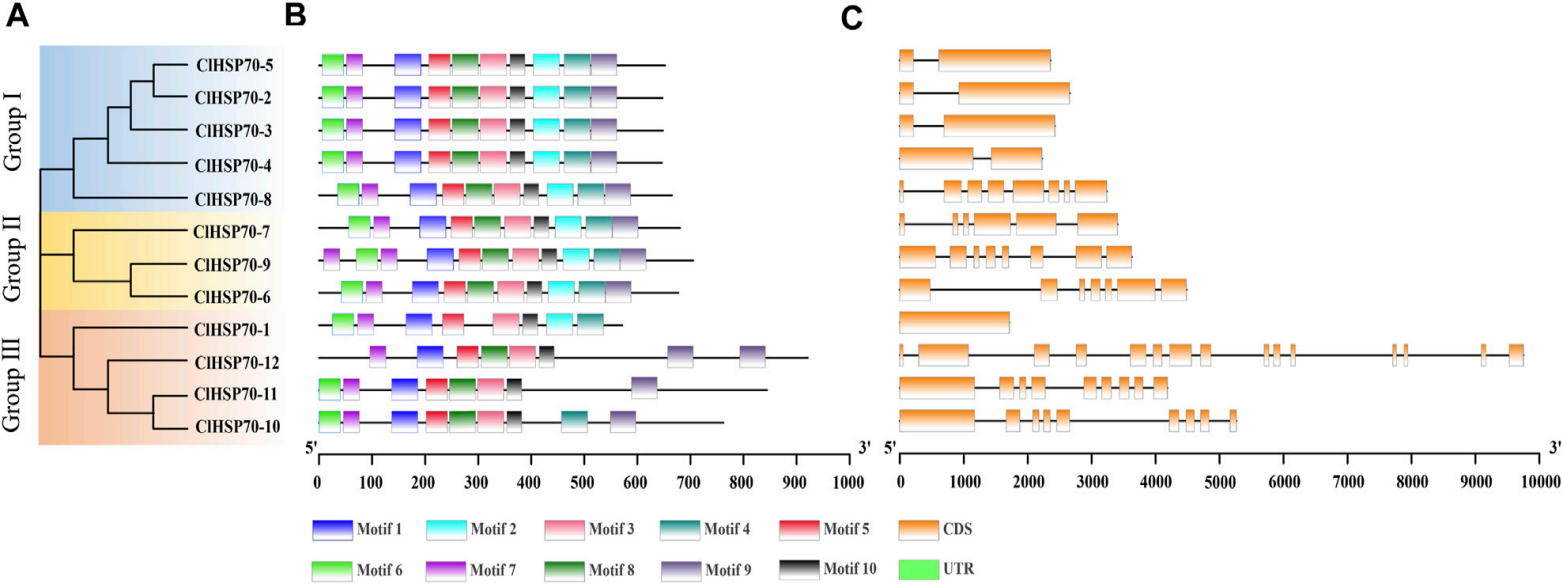
Phylogenetic relationships, gene structures and conserved motifs of all *ClHSP70s* in watermelon. **(A)** The unrooted phylogenetic tree was generated based on the amino acid sequences by the neighbor-joining (NJ) method using MEGA11. Bootstrap supports from 1000 replicates were indicated at each branch. **(B)** Motif analysis was performed using MEME 4.0 software. Different colored boxes represented different motifs in the corresponding position of each ClHSP70 protein. The sequences of different motifs were shown in Supplementary Table S6. **(C)** Gene structures were analyzed using TBtools. Yellow boxes indicated exons, and lines indicated introns.

In addition, the intron number of all members was analyzed to gain insight into the structural evolution of *ClHSP70*s (Figure 1). The intron number of *ClHSP70s* ranged from 0 to 14 (Table 1). The intron number in Group Ⅱ and Ⅲ was higher than other groups, but *ClHSP70-1* from Group Ⅲ had no introns. In general, the exon-intron distribution characteristics of *ClHSP70s* in the same group were similar, while the difference in intron number between different groups was large. Motif analysis showed that the types and amounts of the motifs in Group Ⅰ and Ⅱ were similar, while *ClHSP70-10, -11,* and *-12* in Group Ⅲ did not contain Motif 2, and *ClHSP70-11* and *-12* did not contain Motif 4. Unlike other ClHSP70s, *ClHSP70-12* did not yet contain Motif 6 (Figure 1). The 3D protein structures of ClHSP70s were predicted by SWISS-Model (Supplementary Figure S2) and their GMQE values were higher than 0.8. The number of *β* folds in the protein structure of each ClHSP70 member was higher than that of *α* helices, indicating that *β* folds were the main components of ClHSP70s. The protein structure of ClHSP70-1 was quite different from that of other members, mainly attributing to the fact that the number of *α* helices contained in ClHSP70-1 (13) was much lower than that of other members (≥18). This structural difference was consistent with the results of the cluster analysis of ClHSP70-1 (Figure 1A). The remaining protein structures were highly similar, such as *ClHSP70-2, -3* and *-4*. They had the same number of *α* helices, while *ClHSP70-4* had only one more *β* fold than the other two proteins. These results suggest that *ClHSP70s* are evolutionarily conserved and may have functional redundancy, while structural differences indicate functional specificity among different members.

### 3.2 Chromosomal locations and gene duplication of *ClHSP70s*

**FIGURE 2:**
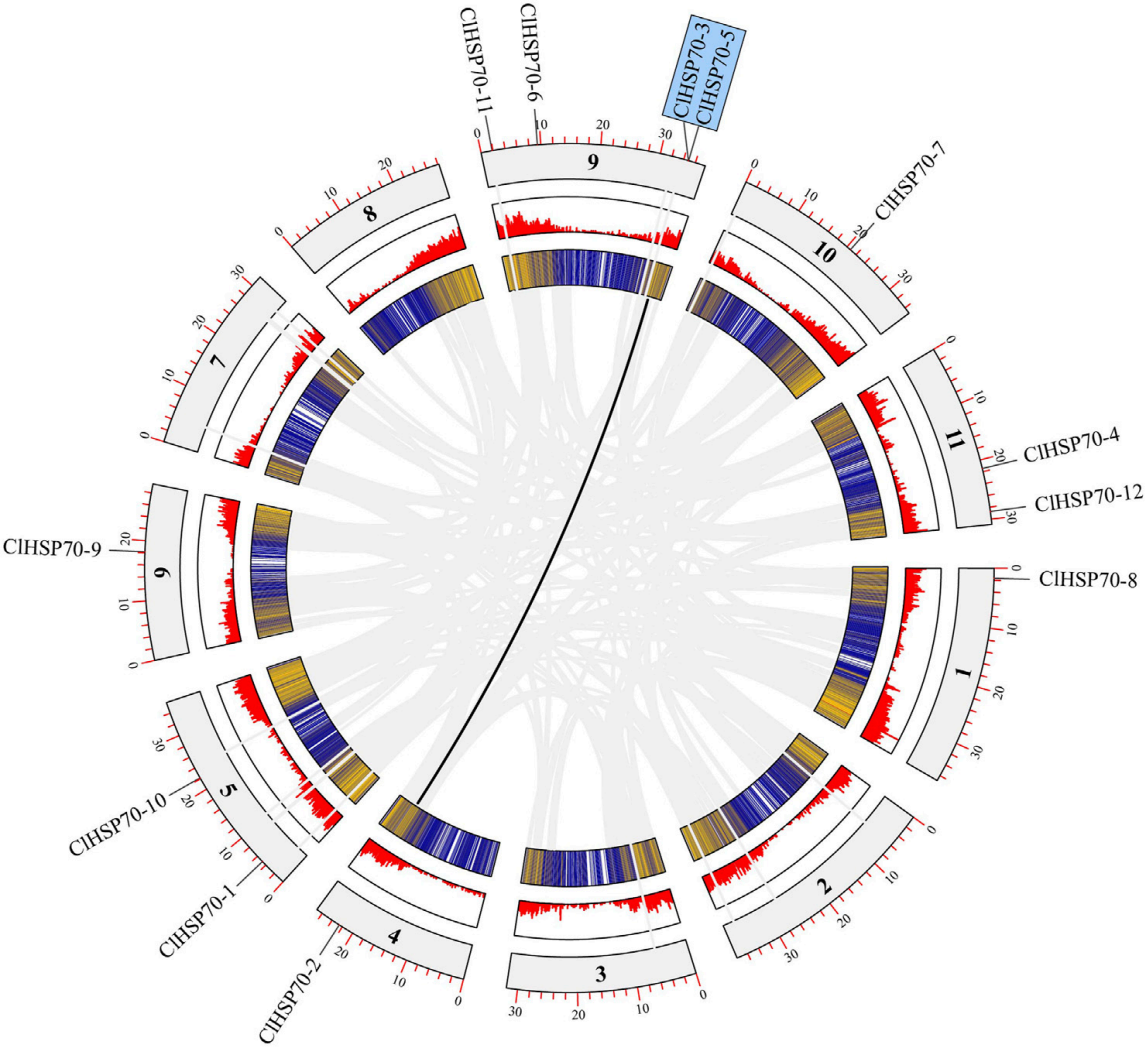
The collinearity analysis of *ClHSP70s*. The outermost circle represented the chromosome, the innermost circle was the gene density in the chromosome, and the middle circle was the gene density histogram display. Grey lines represented all collinearity genes in watermelon, and black lines represented *ClHSP70* collinearity genes.

Based on the annotation file of the watermelon genome, TBtools was used to visualize the distribution of *ClHSP70s* on chromosomes (Chrs). Twelve watermelon *ClHSP70* genes were randomly distributed on 7 Chrs, located on Chr1 (1), Chr4 (1), Chr5 (2), Chr6 (1), Chr9 (4), Chr10 (1) and Chr11 (2), respectively (Figure 2). Tandem duplicated and segmental duplication have certain effects on the formation of gene families during evolution (Cannon et al., 2004). Three pairs of duplication genes in *ClHSP70* family were identified, of which *ClHSP70-3/-5* was tandem duplicated, *ClHSP70-2/-3* and *ClHSP70-2/-5* were segmental duplication (Figure 2). To understand whether *ClHSP70* genes were affected by natural selection during evolution, the Ka/Ks values of *ClHSP70-2/-3*, *ClHSP70-2/-5* and *ClHSP70-3/-5* were calculated. Their Ka/Ks ratios were all less than 1, suggesting that *ClHSP70s* underwent strong purifying selection during evolution (Li et al., 2022), and duplicated events occurred at 123.4 (*ClHSP70-2/-5*), 163.6 (*ClHSP70-3/-5*) and 175.5 (*ClHSP70-2/-3*) Mya, respectively (Table 2).

**TABLE 2:** The divergence between *ClHSP70* gene pairs in watermelon.

Paralogous pairs	Ka	Ks	Ka/Ks	Duplication date (Mya)	Duplicate type
*ClHSP70-2/ClHSP70-3*	0.031	2.142	0.015	175.5	Segmental
*ClHSP70-2/ClHSP70-5*	0.036	1.505	0.024	123.4	Segmental
*ClHSP70-3/ClHSP70-5*	0.038	1.996	0.019	163.6	Tandem

For each gene pair, the Ks value was translated into divergence time in millions of years based on a rate of 6.1 × 10^−9^ substitutions per site per year. The divergence time (T) was calculated as T = Ks/(2 × 6.1×10^−9^) ×10^−6^ Mya.

### 3.3 Phylogenetic analysis of ClHSP70 proteins

**FIGURE 3:**
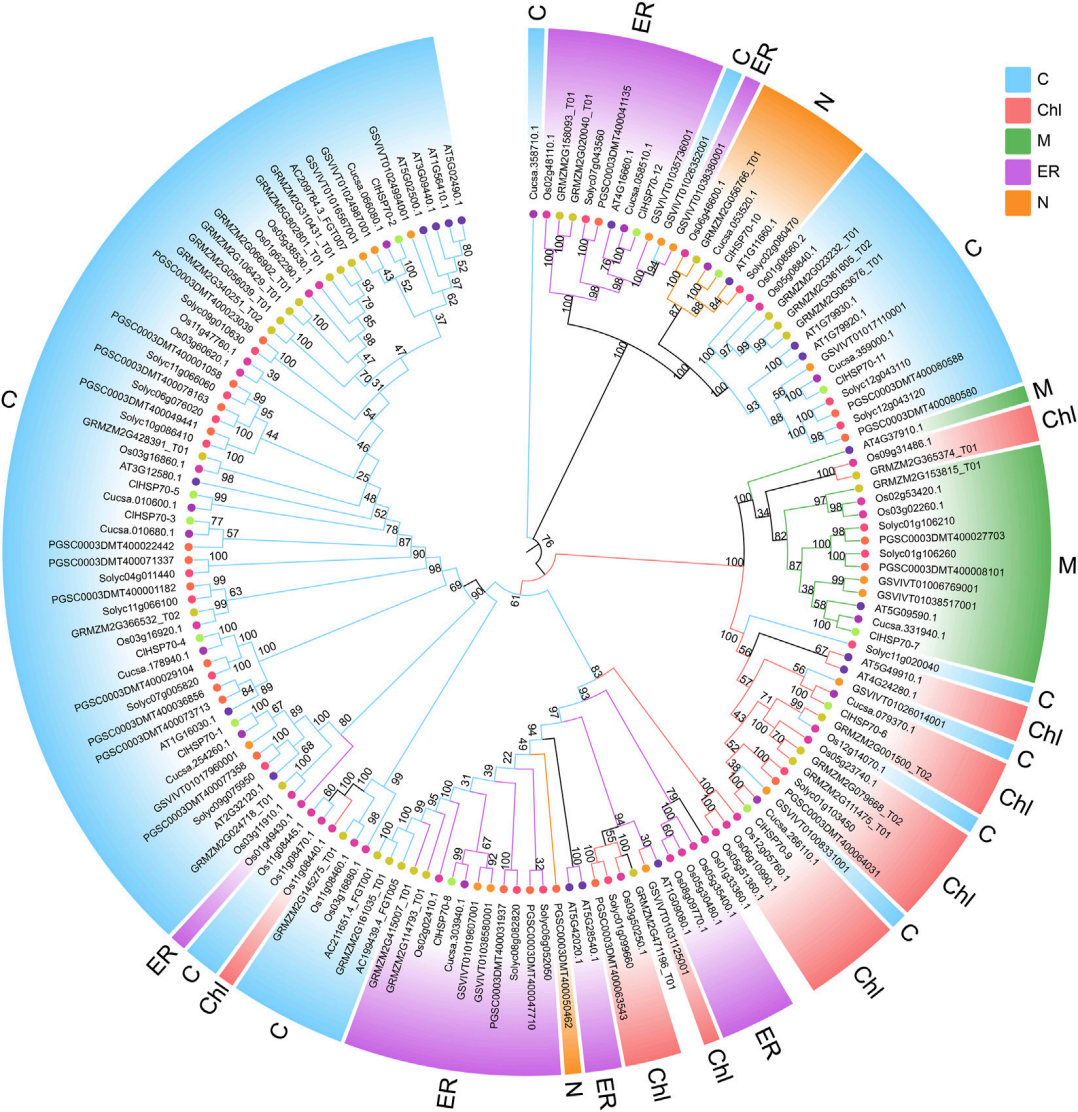
Phylogenetic relationship of ClHSP70 proteins. Phylogenetic trees were constructed using the ML method with bootstrap tests by MEGA11. The diverse *HSP70* subfamilies were indicated with different color arcs. The different colored symbols at the branch tips represented different species. C, cytosol; ER, endoplasmic reticulum; Chl, chloroplast; N, nucleus; M, mitochondria. HSP70 proteins that did not belong to the above subfamilies were not colored.

A ML phylogenetic tree was constructed using the protein sequences of 157 HSP70s from multiple plants, including *A. thaliana* (18), *S. lycopersicum* (19), *S. tuberosum* (21), *C. sativus* (13), *V. vinifera* (15), *Z. mays* (28), *O. sativa* (31), and *C. lanatus* (12) (Figure 3; Supplementary Table S1). Based on their evolutionary relationship, HSP70 proteins were classified into five subfamilies, including cytosol (C), ER, mitochondria (M), chloroplast (Chl) and nucleus (N) subfamily. Nearly half of HSP70s belonged to the cytoplasmic subfamily, which was consistent with the subcellular localization of watermelon *ClHSP70s*, suggesting that plant HSP70s function mainly in the cytoplasm. HSP70 members from different species of the same subfamilies were more closely related than members from different subfamilies. For example, watermelon *ClHSP70-2* and cucumber Cucsa.066080.1 in C subfamily, rice Os06g46600.1 and maize GRMZM2G056766-T01 in N subfamily. Each subfamily had different HSP70 branches formed by monocots and dicots, which also indicated that HSP70 family was formed before the differentiation of monocots and dicots. Twelve pairs of orthologous genes were identified between watermelon and cucumber, suggesting that the ancestral genes of this family were formed before these two species. Additionally, there were several pairs of paralogous genes, indicating that *ClHSP70s* had undergone multiple replications after watermelon speciation.

### 3.4 Interspecific collinearity analysis and *cis*-acting element prediction of *ClHSP70s*

**FIGURE 4:**
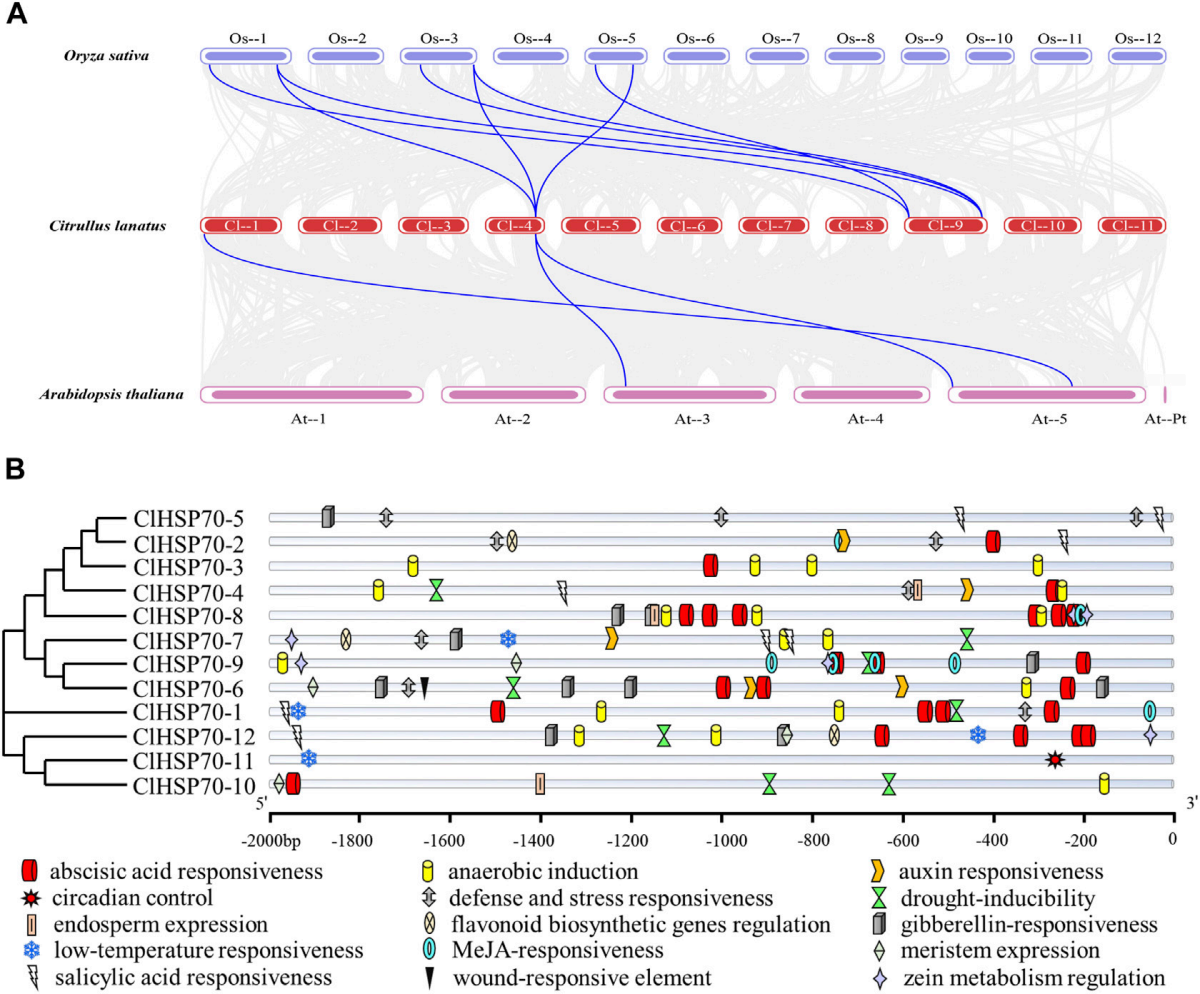
Collinearity and promoter sequence analysis of *ClHSP70* genes. **(A)** Collinearity analysis of watermelon *ClHSP70* genes with *Oryza sativa* and *Arabidopsis thaliana*. The gray lines denoted collinearity between all genes and the blue lines denoted collinearity between the two *ClHSP70* family members. **(B)** Types and numbers of *cis*-acting elements in promoters of watermelon in the *ClHSP70* gene family.

To explore the underlying evolution of *ClHSP70* family, the collinearity of watermelon with model plants *Arabidopsis* and monocotyledon rice was analyzed (Figure 4A). Three pairs of homologous gene pairs between watermelon and *Arabidopsis* (*ClHSP70-2/AtHSP70-2*, *ClHSP70-2/AtHSP70-3*, and *ClHSP70-8/AtHSP70-12*) were identified (Lin et al., 2001), which was much less than 8 pairs between watermelon and rice (*ClHSP70-2/OscHSP70-1*, *ClHSP70-2/OscHSP70-5*, *ClHSP70-2/OscHSP70-6*, *ClHSP70-3/OscHSP70-1*, *ClHSP70-3/OscHSP70-2*, *ClHSP70-3/OscHSP70-5*, *ClHSP70-11/OsHSP110-1*, and *ClHSP70-11/OsHSP110-4*) (Jung et al., 2013).

To investigate the expression characteristics and potential functions of *ClHSP70* family, analysis of the gene promoter sequences (2,000 bp upstream of the start codon) was performed (Supplementary Table S7). The distributions of *cis*-acting elements of *ClHSP70* promoters were visualized, and 15 functional elements were labeled (Figure 4B). The identified *cis*-elements were divided into hormone response group, stress response group, and growth and development group. Among them, the hormone response group contained the most elements, up to 66, mainly including ABA (24), methyl jasmonic (15), gibberellin (11), and salicylic (8). The stress response group also had many *cis*-elements, such as anaerobic induction (19), defense and stress (9), drought stress (8), and low-temperature stress (4). However, only 14 elements in the growth and development group. In summary, *ClHSP70s* may present different expression patterns during developmental and environmental stress response.

### 3.5 Protein interaction network of ClHSP70 family

**FIGURE 5:**
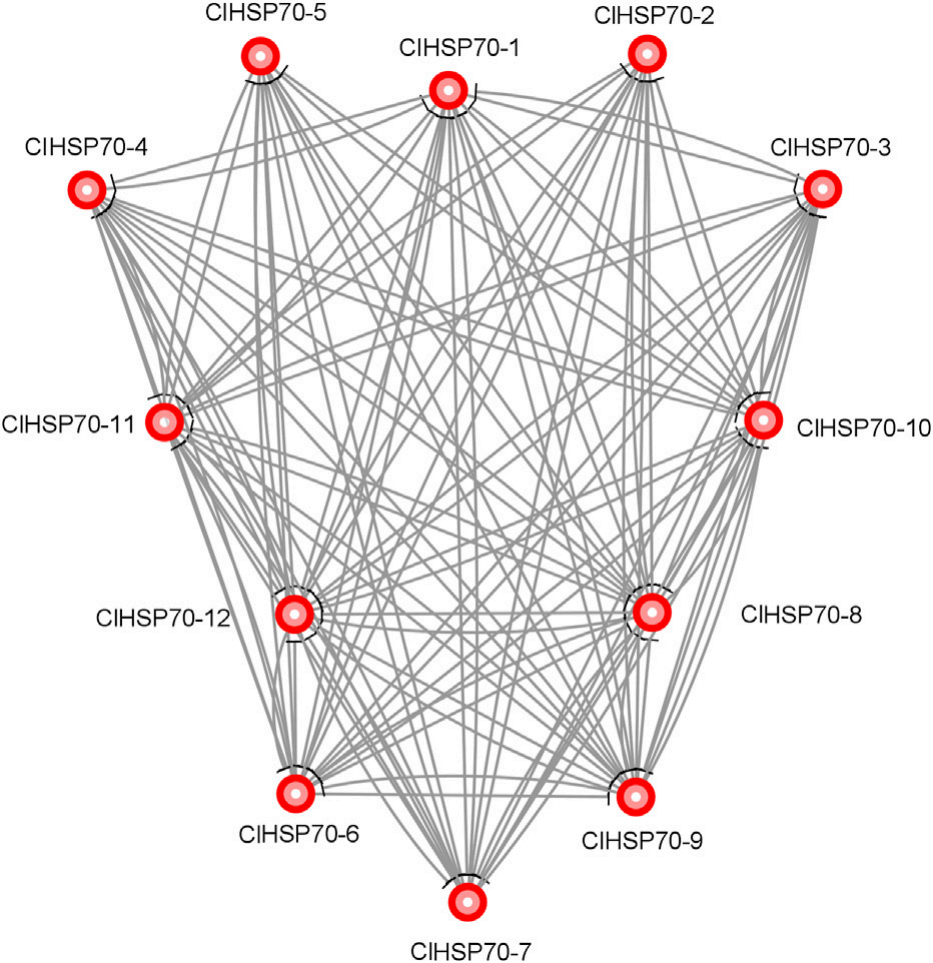
Protein interaction network of watermelon ClHSP70s.

To provide more clues about the function of ClHSP70s, STRING online software was used to predict the protein interaction network of this family members based on the interolog of the *Arabidopsis* interactome. ClHSP70-8 and -12 both interacted with other proteins, while ClHSP70-2 and -5 did not interact with each other, and neither of them interacted with other three proteins (ClHSP70-1, -3, and -4). Additionally, ClHSP70-7 did not interact with ClHSP70-6 or -9 (Figure 5). These data provide a reference for in-depth study of the biological function of ClHSP70s.

### 3.6 GO enrichment and SSR analysis of *ClHSP70s*

In order to deeply understand the functions of this family, GO annotation and enrichment analysis of *ClHSP70s* were performed (Supplementary Figure S3). In MF, all *ClHSP70s* were assigned in binding, such as unfold protein, small molecule, anion and ATP binding. However, only 3 *ClHSP70s* (25%) were assigned in unfold protein binding. In CC, cellular component was enriched in all 12 genes. Nevertheless, only three members (25%) were assigned in protein folding of BP. These data suggest that ClHSP70s may function as molecular chaperones in cells.

A total of 66 SSRs were discovered from 12 *ClHSP70* sequences (Supplementary Table S8). Only the *ClHSP70-9* sequence had one SSR, and the remaining members all had multiple SSRs. In addition, 3 SSRs present in compound formation were detected in *ClHSP70-6*, *-11* and *-12* sequences. The 2000 bp promoter regions contained 23 SSRs, the gene regions contained 19 SSRs, the post-2000 bp sequences contained 24 SSRs, and ClHSP70-6 contained the most SSR sites (14) (Supplementary Table S8). Among all the SSR types, dinucleotide repeats were the most abundant (53.33%), followed by trinucleotide repeats (40.00%), while there was only one SSR (6.67%) with a tetranucleotide repeat. SSRs with five sequence repetitions (26.67%) were the most abundant (Supplementary Table S9).

### 3.7 Tissue-specific expression analysis of watermelon *ClHSP70s*

**FIGURE 6:**
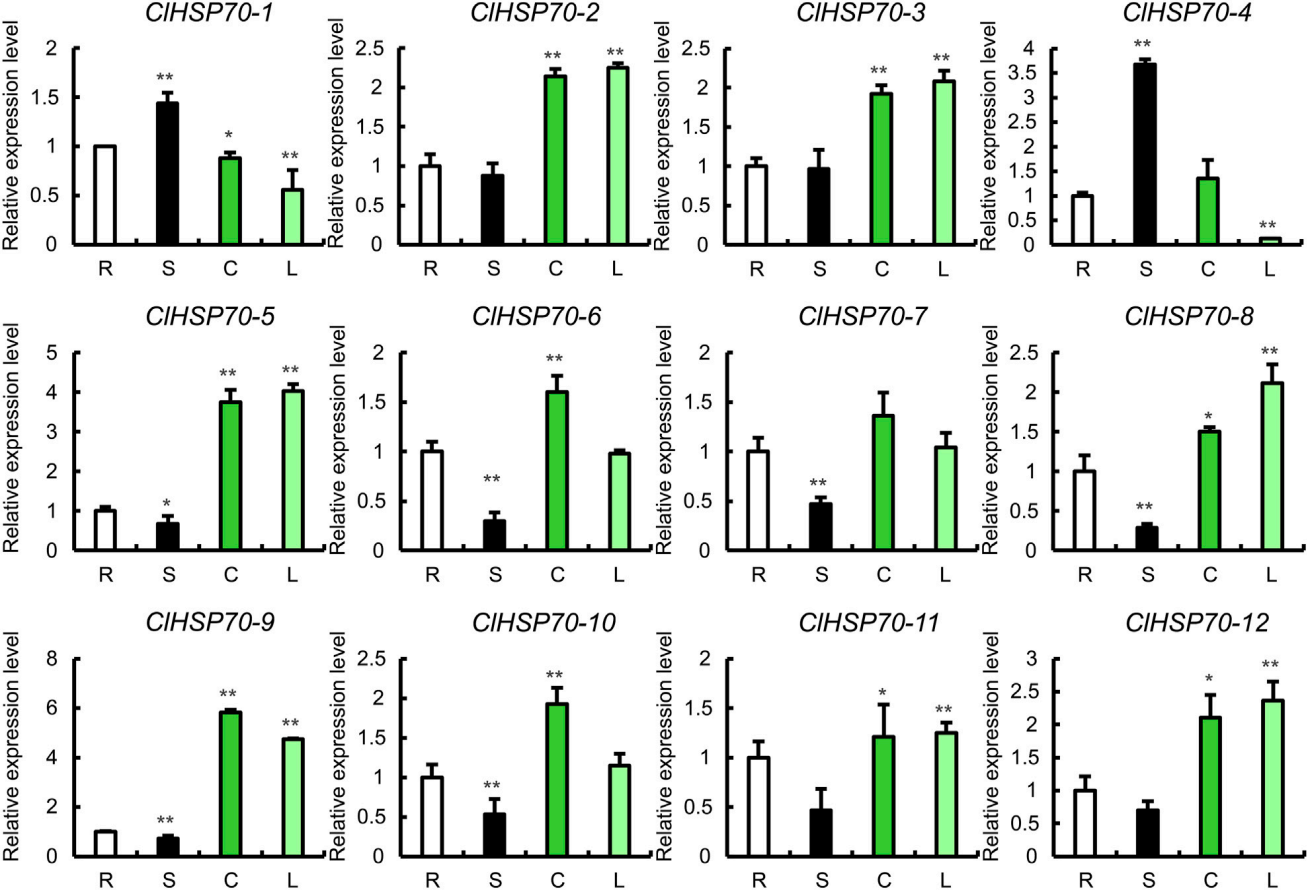
The expression of the *ClHSP70* family genes in different tissues. R, S, C, and L represented roots, stems, true leaves, and cotyledons, respectively. qRT-PCR data were normalized using the watermelon *ClACT* gene. The relative expression levels of genes in roots were set to 1. All statistical tests were two-tailed Student’s *t*-tests at the 0.05 and 0.01 levels, and the *p*-values were given by the symbols * (*p* < 0.05) and ** (*p* < 0.01), respectively.

To analyze the tissue expression specificity of *ClHSP70s*, the relative levels of all members in 4 tissues (roots, stems, true leaves and cotyledons) of 30-day-old seedlings were determined by qRT-PCR (Figure 6). The expression levels of *ClHSP70-1* and *-4* were the highest in stems and the lowest in true leaves. The levels of *ClHSP70-2*, *-3*, *-11* and *-12* in stems were similar to those in roots, but significantly increased in cotyledons and true leaves. The abundance of other six genes (*ClHSP70-5*, *-6*, *-7*, *-8*, *-9*, and *-10*) in stems was the lowest. The expression levels of *ClHSP70-5*, *-8* and *-9* in cotyledons and true leaves were significantly higher than those in other tissues, while *ClHSP70-6* and *-10* were preferentially expressed in cotyledons. In general, *ClHSP70s* may function in the biological processes of different tissues in watermelon.

### 3.8 Response of *ClHSP70s* to drought and cold stress

**FIGURE 7:**
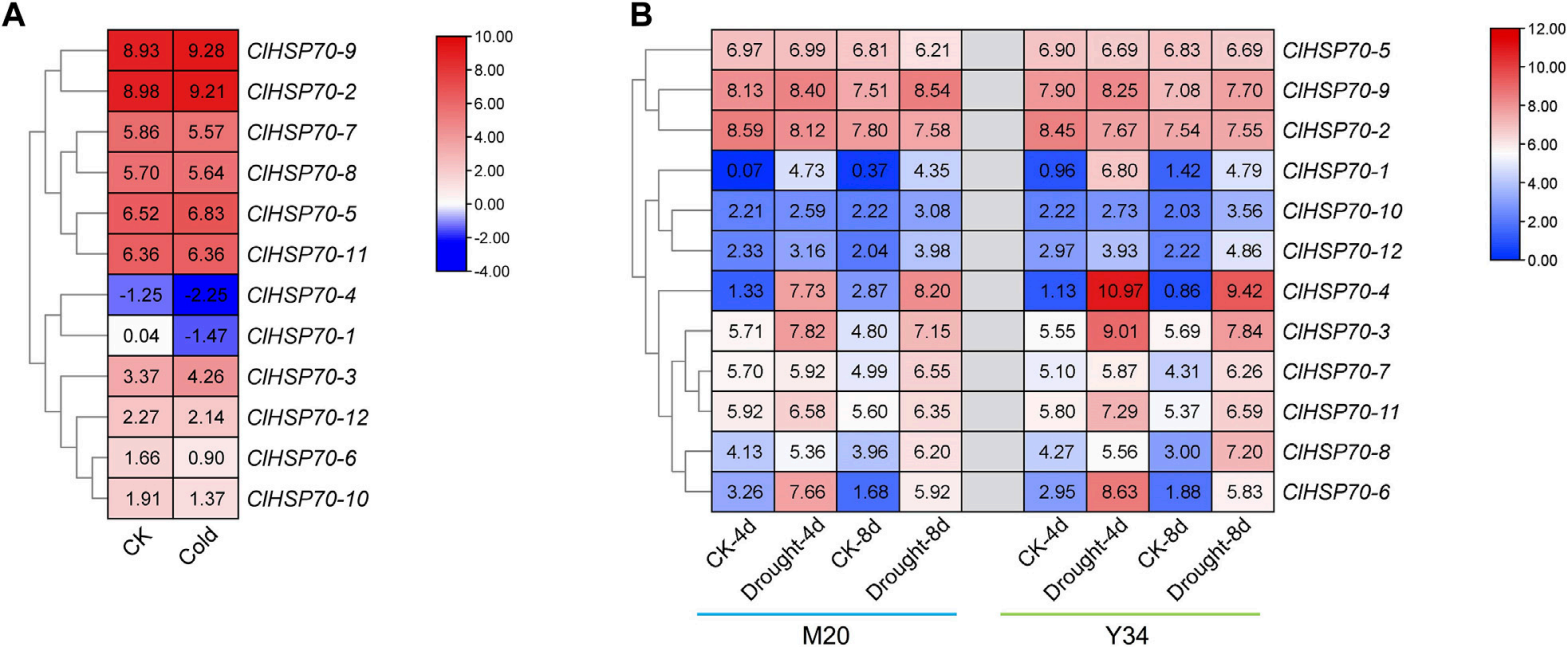
The expression patterns of the *ClHSP70s* under cold stress and drought stress in watermelon. **(A)** The expression of *ClHSP70s* in response to cold stress in Y134. **(B)** The expression of *ClHSP70s* in response to drought stress in M20 and Y34. CK, control; Cold, the seedlings were treated with 4°C for 36 h; Drought, seedlings imposed to drought stress for 4 days and 8 days. The expression of *ClHSP70s* were shown on a heatmap using log2FPKM value, and −4.00 to 10.00 **(A)** and 0.00 to 12.00 **(B)** were artificially set with the color scale limits according to the normalized value.

HSP70s as molecular chaperones are widely involved in plant heat adaptation, and their roles in other abiotic stresses (such as drought and cold stress) have also been demonstrated in a variety of plants (Wang et al., 2004). However, the response of watermelon *ClHSP70s* to drought and cold stress remains unknown. To understand the response of ClHSP70 to cold and drought stress in detail, the effects of cold and drought stress on *ClHSP70* expression were analyzed based on the RNA-seq data from the CuGenDB database (Figure 7). Under cold conditions, all *ClHSP70s* except *ClHSP70-11* responded to low temperature (4°C for 36 h) to varying degrees. In particular, the levels of *ClHSP70-1* and *-4* were significantly downregulated (Figure 7A). Three genes (*ClHSP70-2*, *-5*, and *-9*) were not sensitive to drought stress, however, the levels of other nine members were observably regulated by drought treatment (4 or 8 days) (Figure 7B).

**FIGURE 8:**
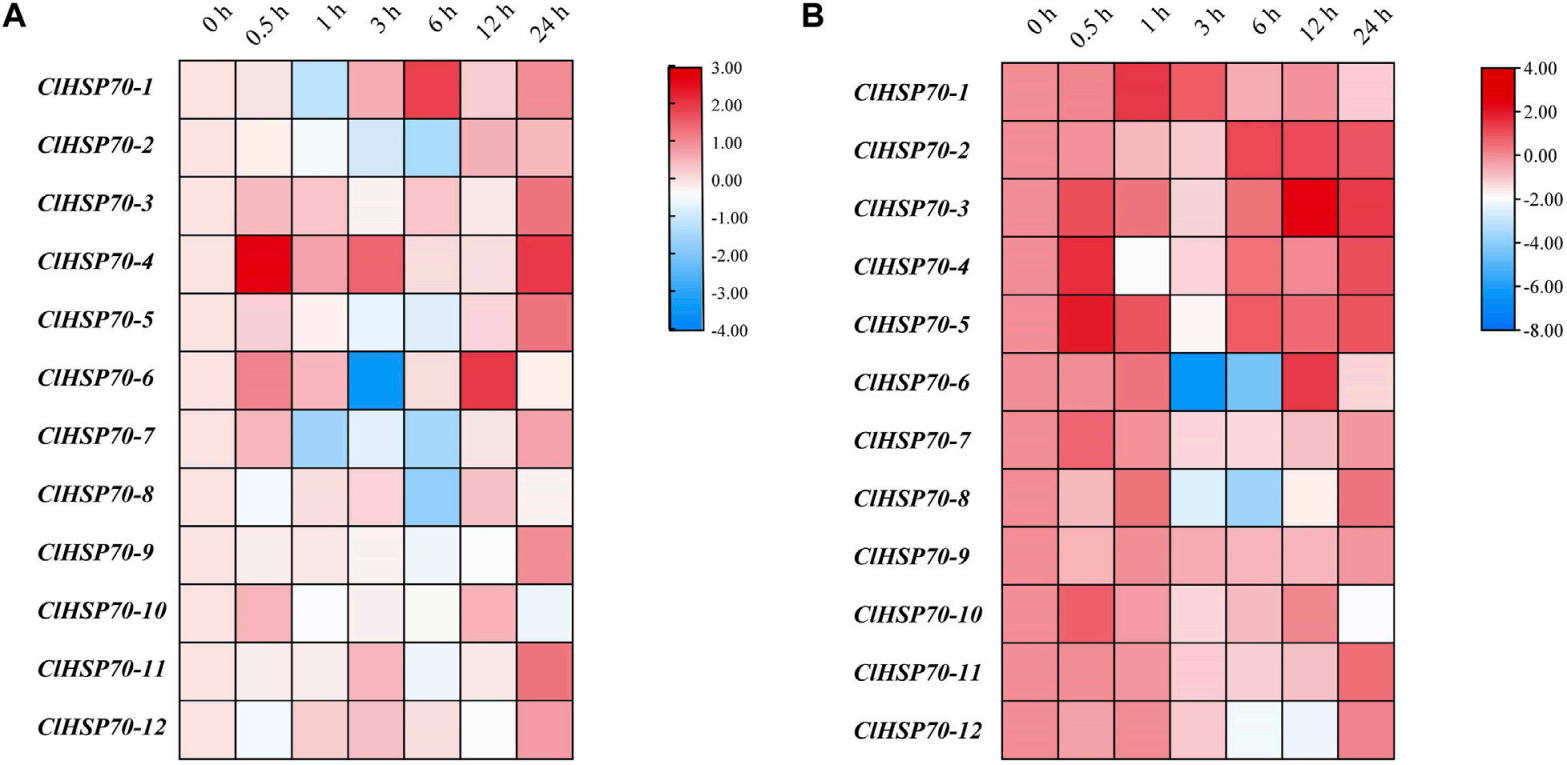
Expression profiles of *ClHSP70* genes in response to drought and cold stress. qRT-PCR analysis of relative *ClHSP70* transcript levels in watermelon plants exposed to drought stress **(A)** and cold stress **(B)**. Drought stress, 30-day-old seedlings treated with 10% PEG6000 for 24 h; cold stress, 30-day-old seedlings treated with 4°C for 24 h. Blocks with blue colors indicated the decreased and red ones indicated the increased transcription levels. qRT-PCR data were normalized using the watermelon *ClYLS8* and *ClACT* genes. The expression levels were relative to that of the corresponding control (seedlings without treatment at the same time). The relative expression data were log2 transformed. The heat maps were created with TBtools.

Moreover, the dynamic expression patterns of all members in 30-day-old seedlings were analyzed under simulated drought (10% PEG6000) and cold stress (4°C) conditions (treatment for 0, 0.5, 1, 3, 6, 12, and 24 h). As shown in Figure 8A, the expression levels of all *ClHSP70s* except *ClHSP70-1* and *-4* were higher after 12 h and/or 24 h of simulated drought treatment. The expression level of *ClHSP70-1* was the highest after drought treatment for 6 h, and the level of *ClHSP70-4* reached the peak after treatment for 0.5 h. Notably, *ClHSP70-1* and *-7* were significantly downregulated after 1 h, the levels of *ClHSP70-2* and *-8* reached the lowest after 6 h, while the lowest level of *ClHSP70-6* was found after 3 h. The expression of *ClHSP70-1*, *-3*, *-4*, *-5*, *-7*, *-8* and *-10* were significantly upregulated by short-term (0.5 or 1 h) cold treatment (Figure 8B). Prolonged treatment inhibited the transcription of many genes to varying degrees. For example, both *ClHSP70-6* and *-8* were significantly downregulated after 3 and 6 h, while the lowest level of *ClHSP70-12* appeared after 12 h. The levels of *ClHSP70-2* and *-3* at 12 and 24 h of treatment were higher than those at other time periods. Different from other *ClHSP70s* regulated by low temperature, *ClHSP70-9* was not sensitive to cold stress. In summary, *ClHSP70s* may function in the formation of drought and cold stress response networks in watermelon.

### 3.9 Response of *ClHSP70s* to exogenous ABA

**FIGURE 9:**
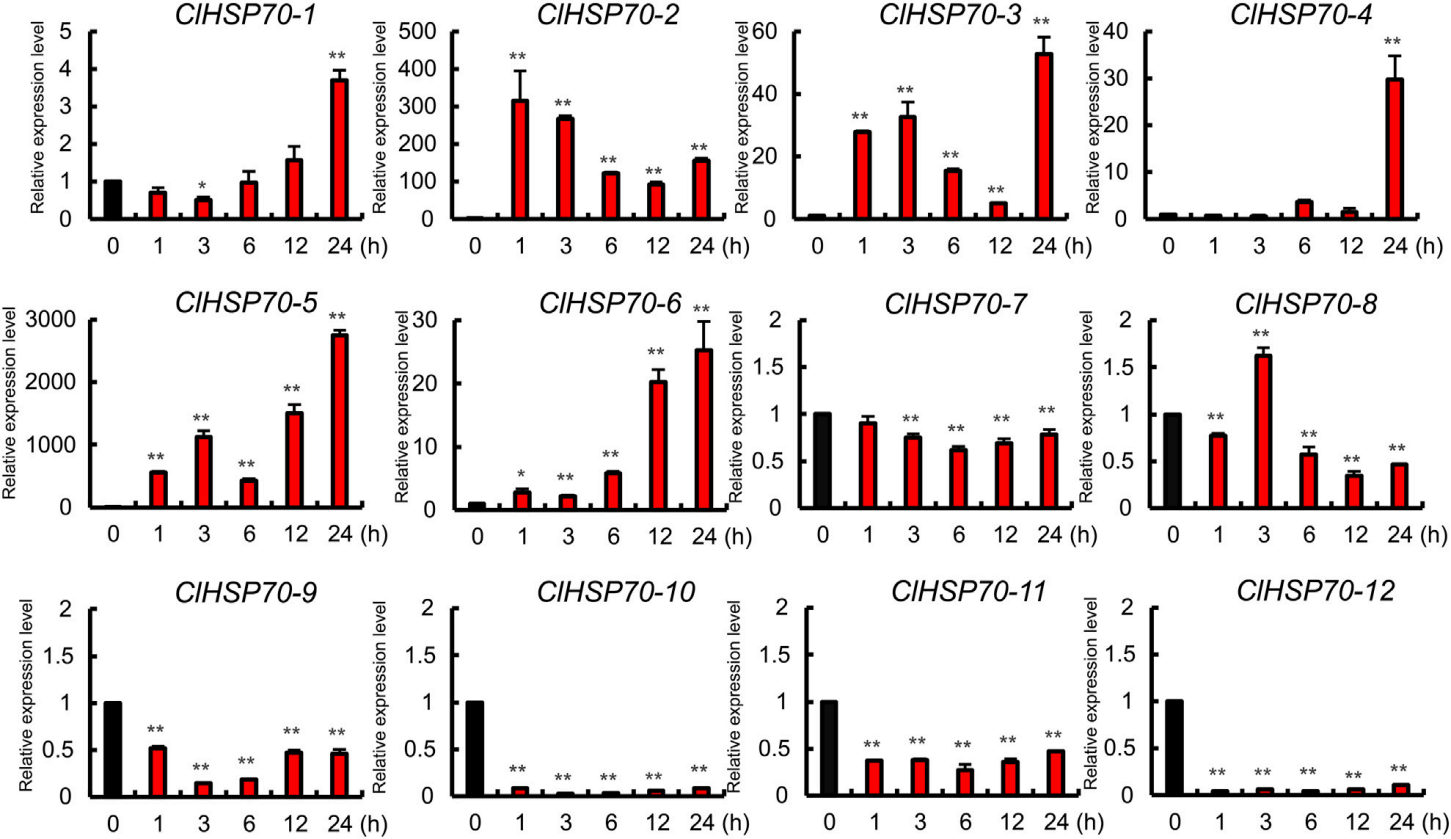
Expression analysis of *ClHSP70* genes in response to ABA. qRT-PCR data were normalized using the watermelon *ClACT* gene. ABA treatment, 30-day-old seedlings were sprayed with 100 μM ABA, and the expression levels of *ClHSP70s* in leaves at different times after treatment were tested. The expression levels were relative to that of the corresponding control (seedlings without treatment at the same time). All statistical tests were two-tailed Student’s t-tests at the 0.05 and 0.01 levels, and the *p*-values are given by the symbols * (*p* < 0.05) and ** (*p* < 0.01), respectively.

ABA signaling pathway is involved in various stresses. The promoters of *ClHSP70s* contained many ABA-responsive elements, suggesting that the expression of these genes may be regulated by ABA. Therefore, 30-day-old watermelon seedlings were treated with exogenous ABA (100 μM), and the expression patterns of *ClHSP70s* in leaves at different times after treatment (0, 1, 3, 6, 12, and 24 h) were analyzed. As shown in Figure 9, the levels of five members (*ClHSP70-7*, *-9*, *-10*, *-11*, and *-12*) were downregulated by ABA, while the remaining seven genes (*ClHSP70-1*, *-2*, *-3*, *-4*, *-5*, *-6*, and *-8*) were upregulated at specific time points. The expression patterns of *ClHSP70-1* and *-6* were similar. Their levels increased gradually with the extension of post-treatment time and reached the highest at 24 h after treatment, which increased 2.7- and 24-fold, respectively. The response of *ClHSP70-4* to ABA was relatively slow, and its expression level increased significantly (nearly 30-fold) only at 24 h after treatment. On the contrary, *ClHSP70-2*, *-3* and *-5* responded rapidly to ABA, and were strongly induced at 1 h after treatment, which increased by about 300-, 30- and 500-fold, respectively (Figure 9). These data suggest that watermelon *ClHSP70s* may be widely involved in ABA-mediated regulation of abiotic stresses.

### 3.10 Analysis of the binding site of *ClHSP70s* targeted by miRNA in watermelon

**FIGURE 10:**
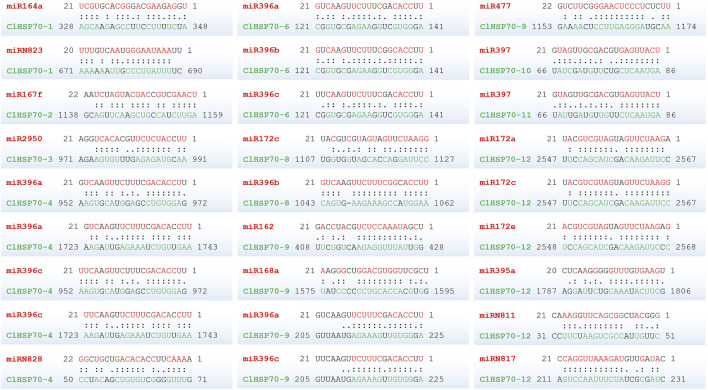
The predicted binding site of the *ClHSP70s* targeted by miRNAs in watermelon. Two dots indicated paired successfully between bases, and one dot indicated that there was also a pairing between U and G in the secondary structure. Blank space indicated that two bases failed to be paired. miRNAs and their paired bases were marked in red, and *ClHSP70s* and their paired bases were marked in green.

To determine whether *ClHSP70s* were regulated by microRNAs (miRNAs) through targeted binding sites, the binding sites of miRNAs targeting *ClHSP70s* were predicted. As shown in Figure 10, 10 *ClHSP70s* (*ClHSP70-1*, *-2*, *-3*, *-4*, *-6*, *-8*, *-9*, *-10*, *-11*, and *-12*) might be regulated by 28 miRNAs. Among them, four genes (*ClHSP70-2*, *-3*, *-10*, and *-11*) contained only one miRNA binding site, while the binding sites of *ClHSP70-12* targeted by miRNA was the most. Notably, among the five binding sites regulating *ClHSP70-4*, two sites each were targeted by miR396a and miR396c, and one was targeted by miRN828. Furthermore, both miR396a and miR396c could regulate *ClHSP70-6* and *-9*, and miR397 regulated *ClHSP70-10* and *-11* (Figure 10; Supplementary Table S4). These results indicate that one miRNA can regulate multiple *ClHSP70s* and *vice versa*.

## 4 Discussion

Abiotic stresses adversely affect the growth and development of plants and lead to a significant decrease in crop yield. The molecular chaperone HSP70 proteins can enhance stress tolerance in plants (Saeed et al., 2019). Therefore, a comprehensive analysis of HSP70 family in plants is of great biological significance. Characterizing the HSP70 family at the genome-wide level has been performed in a variety of plants, such as *Arabidopsis*, tomato, pumpkin, grape, maize, and rice (Sung et al., 2001; Sarkar et al., 2013; Jiang et al., 2021; Abbas et al., 2022; Davoudi et al., 2022; Liu et al., 2022). However, this family in watermelon has not been systematically studied.

In this study, 12 *ClHSP70s* were identified in watermelon, which was similar to the number in cucumber (13) and grapevine (15), but less than that in tomato (19), maize (28) and rice (31) (Figure 3; Supplementary Table S1). This difference may be related to their genome sizes or evolutionary diversities (Sung et al., 2001; Zhang et al., 2015; Guo et al., 2016; Kang et al., 2022). Gene structure and phylogenetic tree were helpful to analyze the evolutionary relationship among genes(Wang et al., 2019). Genes closely related to phylogeny usually have similar properties or functions, and are generally consistent in subcellular locations (Cho and Choi, 2009; Kose et al., 2012). The Group I members of watermelon *ClHSP70* family, such as *ClHSP70-2*, *-3*, *-4* and *-5*, were closely related in evolution and had almost the same gene structure. All of them contained one intron and two exons, and the proteins encoding by these genes were localized in cytoplasm, indicating that they may have the similar function. The number of introns was generally affected by transcriptional regulation (Yu et al., 2021). Genes with many introns generally respond slowly to stresses, while genes with a small number of introns were sensitive to stresses (Jeffares et al., 2008; Jin et al., 2014). The distributions of exons among *ClHSP70s* in Group Ⅰ, Ⅱ, and Ⅲ were different, while the difference within the group was not significant (Figure 1), which was consistent with that in tea plants (Chen et al., 2018). Phylogenetic analysis revealed that watermelon ClHSP70 family was more closely related to cucumber than to *Arabidopsis* (Figure 3), as well as the results of pumpkin HSP70 family (Davoudi et al., 2022), indicating that HSP70 family is conserved during evolution, but also exhibits species specificity.

Gene duplication events not only support the functional evolution of genes but also innovate their structures, which is crucial to the evolution of gene families (Moore and Purugganam, 2003; Kong et al., 2007). Factors such as polyploidy and segmental and tandem duplication have extended the number of plant gene families and mutations in the regulatory regions of new members probably cloud modify the expression patterns and function of duplicated genes (Hashemipetroudi et al., 2023; Yaghobi and Heidari, 2023). Two or more adjacent gene duplications on the same chromosome were considered tandem duplications, while gene duplications on two different chromosomes were considered segmental duplications (Cannon et al., 2004). Collinear analysis identified two pairs of segmental repeats (*ClHSP70-2/-3* and *ClHSP70-2/-5*) and a pair of tandem repeats (*ClHSP70-3/-5*) (Figure 2), which was consistent with the finding that segmental duplication was more than tandem duplication in soybean families (Schlueter et al., 2007). The Ka/Ks ratios of all gene pairs were less than 1 (Table 2), suggesting that this species had a strong Darwinian positive evolutionary selection as early as 1.7 Mya (Omidbakhshfard et al., 2015).

Plant *HSP* genes, such as *HSP20s*, *HSP70s* and *HSP90s*, have obvious tissue specificity in various species (Wehmeyer and Vierling, 2000; Wang et al., 2004; Liu et al., 2018). For example, the levels of some *Arabidopsis AtHSP90s* were lower in stems and flowers, but higher in roots, while *AtHSP90-1* only expressed in roots (Yabe et al., 1994). In *Populus*, except for *PtHSP90-5a* and *-5b*, *PtHSP90* genes were mainly expressed in stems (Zhang et al., 2013). Moreover, the expression patterns of tomato *SlHSP20s* and tea *CsHSP70s* showed obvious tissue specificity (Yu et al., 2016; Chen et al., 2018). Similarly, the expression of watermelon *ClHSP70s* was also significantly different in multiple tissues (Figure 6). For instance, *ClHSP70-4* highly expressed in stems, while its level in cotyledons was low or even absent, revealing that different *ClHSP70s* may function in specific tissues. Interestingly, *ClHSP70-2*/*-3*, *ClHSP70-2*/*-5* and *ClHSP70-3*/*-5* homologous genes pairs had similar expression patterns in different tissues. Their levels in true leaves and cotyledons were significantly higher than other two tissues, which was similar to the results in soybean (Cho and Hong, 2006). However, the expression patterns of homologous gene pairs were not completely consistent. For example, the levels of pepper *CaHSP70-11/-16* homologous genes were significantly different in different tissues (Guo et al., 2016).

In addition, *cis*-acting elements in promoters also play central roles in gene expression. The *ClHSP70* promoters mainly contained ABA-response elements (ABREs) and stress response elements (Figure 4B). The leucine zipper transcription factor AREBs usually bind to ABREs in the promoters to regulate downstream genes (Yoshida et al., 2010). ABA was widely involved in abiotic stress response in plants (Wasilewska et al., 2008; Lee and Luan, 2012). It can be speculated that *ClHSP70s* may be regulated by ABA and abiotic stresses. There were 70 ABRE elements in promoters of *N. tabacum NtHSP70s*, most of which were activated by ABA (Song et al., 2019). In this experiment, most *ClHSP70s* were also regulated by ABA (Figure 9). *ClHSP70-2*, *-3*, *-4*, *-5* and *-6* showed ultra-high expression, while the levels of *ClHSP70-10* and *-12* decreased under ABA treatment. This different response to ABA may be closely related to the number and location of ABREs in their promoters. However, the function of ABREs, ABA and AREBs in the expression regulation of watermelon *ClHSP70s* needs further study. Furthermore, SSRs may play an important role in gene regulation (Panzade et al., 2021). In this study, a total of 66 potential SSRs were mined from *ClHSP70s* (Supplementary Table S8). Most SSRs were located in the intergenic region (promoter regions and 3′untranslated regions), which was similar to that in *Ziziphus jujuba* (Panzade et al., 2021). The identification and analysis of SSRs of these genes will help to identify the functions of *ClHSP70s* and enhance the efficiency of stress tolerance breeding in crops (Fu et al., 2023).

Plant *HSP70* genes are involved in various abiotic stress responses and have potential functions in improving resistance to stresses (Alvim et al., 2001). Most tea *CsHSP70* genes had a positive response to drought stress (Chen et al., 2018). Twelve barley *HvHSP70s* were induced after drought stress, and *PtHSP70s* showed different expression patterns in drought-sensitive and -tolerant *Populus* species after drought stress (Zhang et al., 2013; Chaudhary et al., 2019). Based on the analysis of RNA-seq data, watermelon *ClHSP70s* were found to be involved in the response to cold and drought stress (Figure 7). qRT-PCR analysis further found that *ClHSP70-1*, *-4,* and *-6* were strongly induced by PEG-simulated drought treatment (Figure 8A), suggesting that these genes may participate in drought response. In addition, HSP70s also responded to cold stress (Chaudhry and Sidhu, 2022). Most grape *VaHSP70s* showed obvious stress regulation under cold conditions, among which *VaHSP70-19* was continuously highly expressed under cold treatment, whereas other *VaHSP70s* were downregulated (Liu et al., 2022). The levels of *Arabidopsis AtHSP70s* and the chaperone genes were upregulated after cold treatment, while *AtHSP70-16* was negatively regulated in seed germination, which may be a protective mechanism against cold stress (Ashraf et al., 2021). The expression of watermelon *ClHSP70-3*, *-4* and *-5* was upregulated under cold stress, however, *ClHSP70-6* and *-8* were significantly downregulated at 3 and 6 h (Figure 8B). In conclusion, *ClHSP70s* were regulated by ABA, cold and drought stress, suggesting that ClHSP70 family may function in abiotic stress response mediated by ABA.

The heat shock elements (HSEs) in promoters are important for the regulation of corresponding genes under heat stress (Sarkar et al., 2013; Guo et al., 2016). However, no HSEs were identified in *ClHSP70* promoters, consistent with the case of *Pyropia yezoensis PyHSP70s* (Yu et al., 2021). Despite this, *ClHSP70* promoters contained many elements related to stress and hormone response (Figure 4B), implying the potential function of *ClHSP70s* in heat response. In fact, the molecular chaperone function of HSP70s was identified mainly under heat stress conditions (Sung et al., 2001). GO enrichment analysis also showed that some *ClHSP70s* were involved in protein folding and unfolded protein binding (Supplementary Figure S3). ClHSP70s may participate in the heat response of watermelon through indirect ways, such as hormonal signals or cross pathways between different stresses. Identification of ClHSP70s′ functions under high temperature conditions is helpful to clarify the molecular mechanism of heat response, providing theoretical guidance for the genetic improvement of watermelon heat tolerance. However, there may be redundancy in the functions of different members of the ClHSP70 family in heat or other abiotic stress response, making it particularly important to parse the function of each ClHSP70 member. Rapid advances in biotechnology are expected to accelerate this goal. For example, genetic transformation technology can be used to create materials for excessive gene expression, RNA interference (RNAi) or virus induced gene silencing technology (VIGS) can be used to knock down the expression of a single target gene. At the same time, transcriptome sequencing can be used to analyze the effect of specific target gene changes on the expression of other genes in plants under abiotic stresses. The co-expression network constructed using transcriptome data can functionally annotate the regulated genes (Panahi et al., 2019), thus clarifying the roles of watermelon *ClHSP70s* in different biological pathways under abiotic stresses. In addition, gene editing techniques can be used to simultaneously edit multiple members of *ClHSP70* family, creating mutants with multiple genes knocked out, as in tomato (Li et al., 2018). This method not only helps to verify the interaction of different members under abiotic stresses, but also provides new germplasm for genetic improvement of watermelon stress tolerance.

miRNAs are essential modulators of transcriptional gene regulation in plants (Zakeel et al., 2019). Up to now, many plant miRNAs have been identified to respond to different stress conditions. For example, in *Arabidopsis*, miR396 played an important role in response to salt stress (Li et al., 2008) and miRNA397 improved plant tolerance to cold stress (Dong and Pei, 2014). In addition, several studies have revealed that miR396 and miR397 targeted the stress (heat, cold, drought, and salt)-responsive *HSP70* genes in *Brassica rapa* (Tabusam et al., 2022), *Populus* (Yer et al., 2016) and common bean (Büyük et al., 2016). In this study, miR396 targeted *ClHSP70-4*, *-6*, *-8* and *-9*, while miR397 targeted *ClHSP70-10* and *-11*. Many other miRNAs, such as miR164, miR167, miR168, miR172 and miR395, also targeted watermelon *ClHSP70s* (Figure 10; Supplementary Table S4). Therefore, the identification of miRNAs may play a crucial role in understanding the function of *ClHSP70* family genes.

In this study, *ClHSP70* family in watermelon genome was characterized for the first time. A total of 12 genes were identified. We analyzed the physical and chemical properties of this family. The systematic analysis of gene structure, phylogenetic tree, *cis*-acting elements in promoters, protein interactions, SSRs, GO enrichment analysis, binding sites targeted by miRNAs, and collinearity were performed. In addition, the expression patterns of *ClHSP70s* were identified by RNA-seq and qRT-PCR. Watermelon *ClHSP70s* were tissue specific, and had different degrees of response to ABA, drought and cold stress. Our results provide a reference for a better understanding of the functions of ClHSP70 family in watermelon.

## Data Availability

The original contributions presented in the study are included in the article/Supplementary Material, further inquiries can be directed to the corresponding author.
