# Supplementary material for: Characterization of HSP70 family in watermelon (Citrullus lanatus): identification, structure, evolution, and potential function in response to ABA, cold and drought stress
Source: Front Genet. 2023 May 31;14:1201535. doi: 10.3389/fgene.2023.1201535 (PMC10265491; doi:10.3389/fgene.2023.1201535)
Supplement: Supplementary file 2 [file DataSheet1.docx]

**
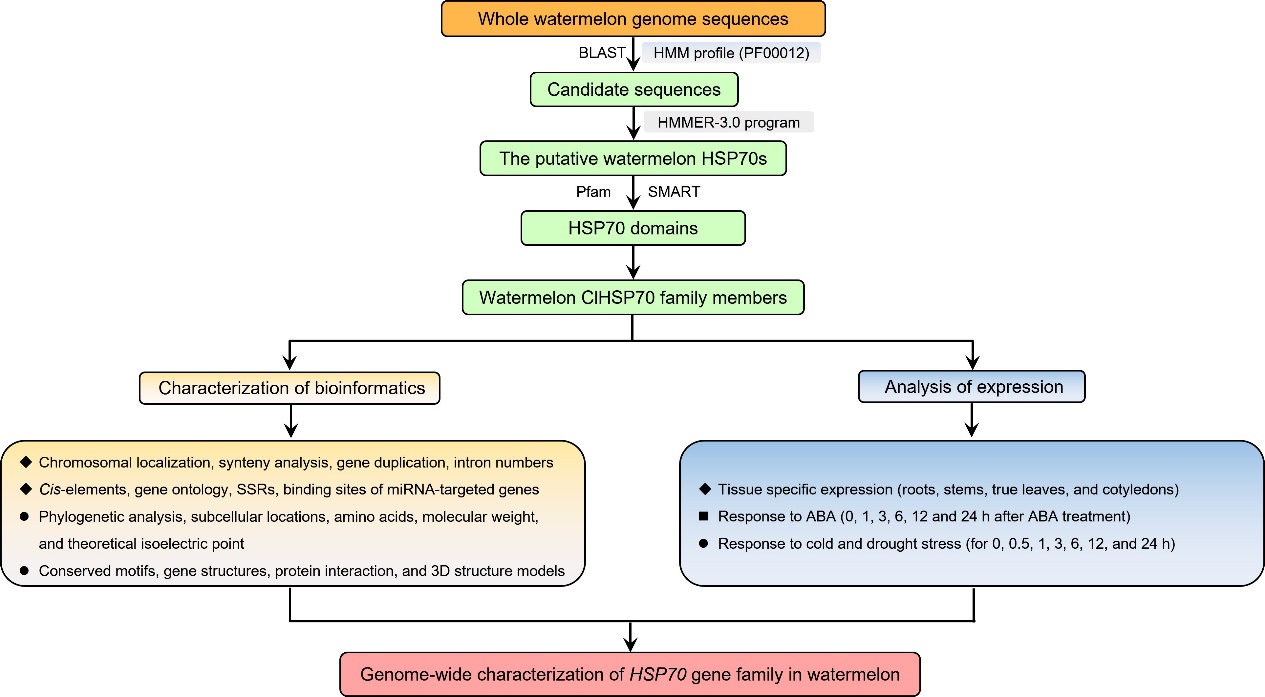
Figure S1.** Flowchart used to identify and characterize the watermelon ClHSP70 family.


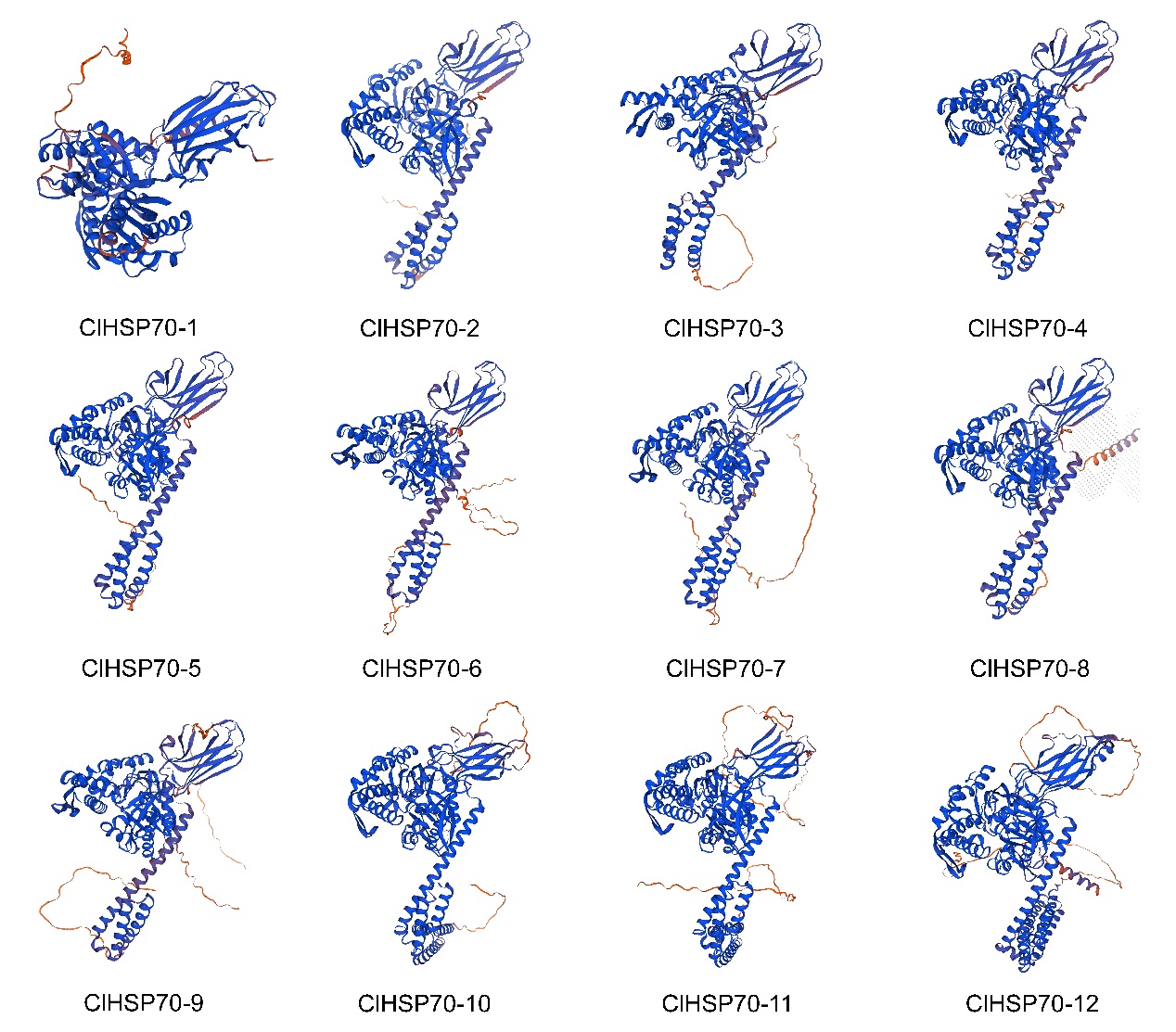
**Figure S2.** Three-dimensional (3D) protein structures of 12 ClHSP70s.

**
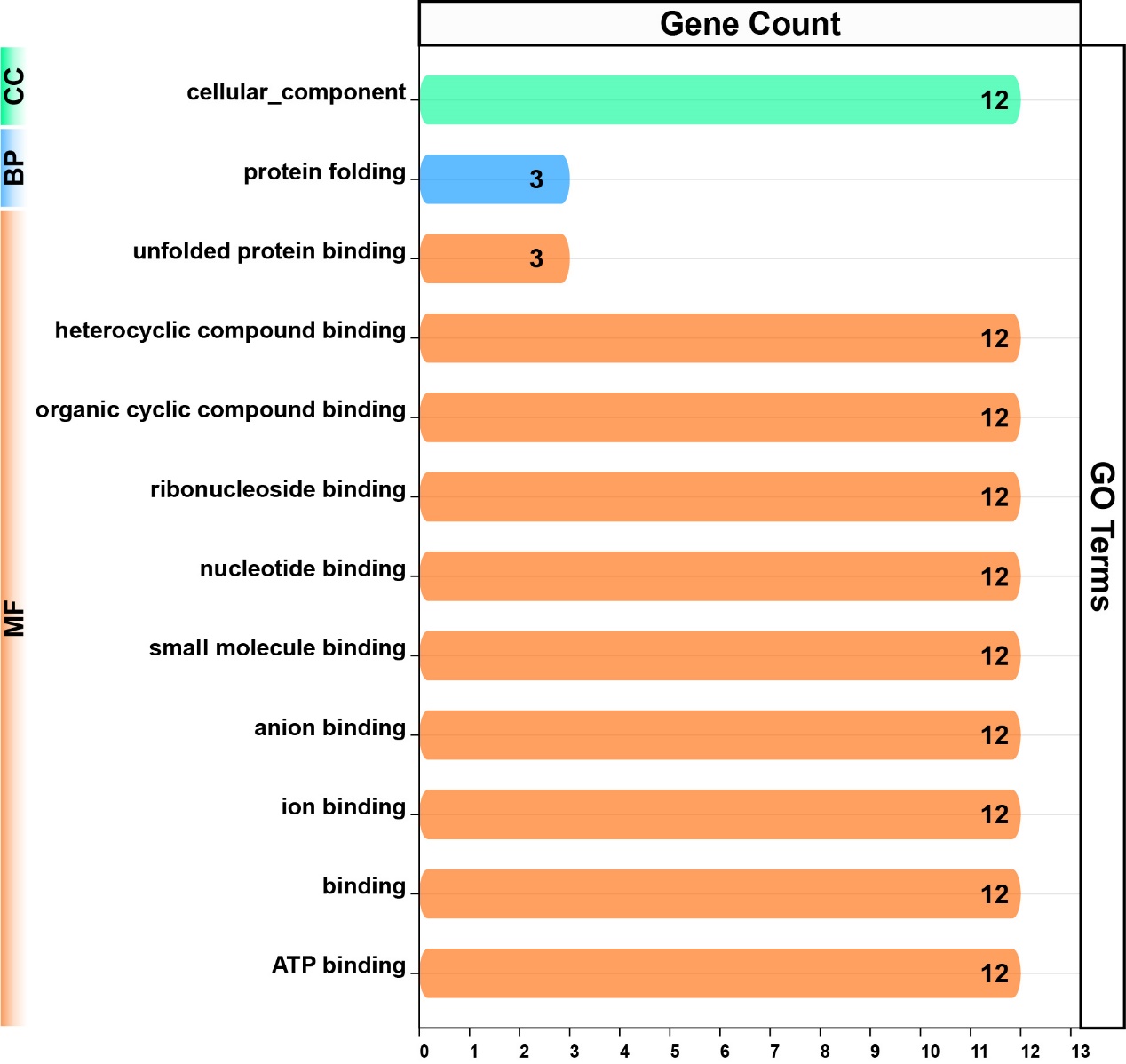
Figure S3.** GO enrichment analysis of *ClHSP70s*. GO was performed with three main categories: molecular function (MF), cellular component (CC) and biological process (BP). GO terms with *p*-value < 0.05 were identified as significant.
